# Severe kyphoscoliosis observed in a human skeleton from the Edo period

**DOI:** 10.1537/ase.250227

**Published:** 2025-05-15

**Authors:** Yasuo Hagihara, Tamotsu Murayama, Akifumi Yoshida, Takashi Nara

**Affiliations:** 1 Institute for Physical Anthropology, Niigata University of Health and Welfare, Niigata, 950-3198 Japan; 2 Department of Physical Therapy, Faculty of Rehabilitation, Niigata University of Health and Welfare, Niigata, 950-3198 Japan; 3 Niizashiki Central General Hospital, Saitama, 352-0001 Japan; 4 Department of Radiological Technology, Faculty of Medical Technology, Niigata University of Health and Welfare, Niigata, 950-3198 Japan

**Keywords:** scoliosis, palaeopathology, Edo, skeleton, kyphoscoliosis

## Abstract

A severe case of kyphoscoliosis was observed in the skeletal remains of a male from the late Edo period in Japan. This individual exhibited left curvature thoracic scoliosis and kyphotic deformity, with ankylosis of the fifth to twelfth thoracic vertebrae at the intervertebral joints. The Cobb angle was 77° and the kyphotic angle was 76°. The superior articular facet of the cervical vertebrae was lower on the left side than the right side. Additionally, the sacral base was inclined to the right, resulting in the superior articular process and facet being lower on the right than on the left. These observations suggest compensatory right curvature of the cervical and lower lumbar spine. The rib morphology was asymmetrical, with the right ribs being obtusely angled and spreading laterally, while the left ribs were acutely angled and directed medially. These findings indicate that this individual had severe structural left-convex thoracic kyphoscoliosis. The possible underlying conditions considered in this case included idiopathic scoliosis or syringomyelia. This is the first detailed report of severe kyphoscoliosis in archaeological skeletal remains excavated from the Japanese Archipelago.

## Introduction

The spine often shows abnormal curvatures such as scoliosis or kyphosis. Scoliosis indicates lateral curvature in the frontal plane, and kyphosis indicates excessive posterior curvature in the sagittal plane. Scoliosis is a three-dimensional structural deformity of the spine that is clinically diagnosed based on a measurement of the spinal curvatures using the Cobb angle when the spine curves laterally by more than 10° in the coronal plane (Cheng et al., 2015). The frequency of scoliosis is estimated to be 2–3% of the population at risk (Weinstein et al., 2008). Spinal deformities can be classified into structural deformities caused by the deformation of the vertebrae themselves, and functional deformities resulting from pain, leg length discrepancy, or abnormal posture (Cheng et al., 2015). Idiopathic scoliosis is the most common cause of structural scoliosis, accounting for approximately 80% of all cases. Other causes include congenital and neuromuscular scoliosis due to conditions such as cerebral palsy, muscular dystrophy, syringomyelia, and mesenchymal tissue disorders such as Marfan syndrome (Appleby et al., 2014; Aufderheide and Rodriguez-Martin, 1998; Waldron, 2021). Structural kyphosis often results from trauma (e.g. fractures) or disease (e.g. Scheuermann’s disease) (Waldron, 2021). Severe scoliosis, also known as kyphoscoliosis, is frequently accompanied by kyphosis (Wang et al., 2012).

Among the abnormal curvatures of the spine, scoliosis has been identified in several archaeological skeletal remains. A notable example is provided by Richard III, the last Plantagenet king of England, in the 15th century (Appleby et al., 2014). It was concluded that Richard III had idiopathic scoliosis based on the absence of any bone deformities associated with any specific disease and the lack of malformed vertebrae. Furthermore, skeletal remains from 15th- to 16th-century Ireland exhibited severe scoliosis along with marked atrophy and shortening of the right lower limb, suggesting that this individual may have had cerebral palsy with right hemiplegia (Tesorieri, 2016). Although there are other reports of scoliosis in ancient skeletal remains (Kilgore and Gerven, 2010; Cirak and Emel, 2023; Umbelino et al., 2012), there are only a few reported cases of this disease despite its wide recognition. This is because vertebrae are less likely to be preserved intact compared with other bones, and diagnosing spinal deformities is difficult when the vertebrae are disarticulated or partial (Aufderheide and Rodriguez-Martin, 1998; Lewis, 2019). Notably, there have been no detailed reports of scoliosis in archaeological skeletal remains from the Japanese Archipelago. The current study aimed to elucidate the details and the underlying causes of pronounced thoracic kyphoscoliosis in male skeletal remains from the late Edo period, through macroscopic observation and morphometric analysis.

## Materials and Methods

**Figure 1.**
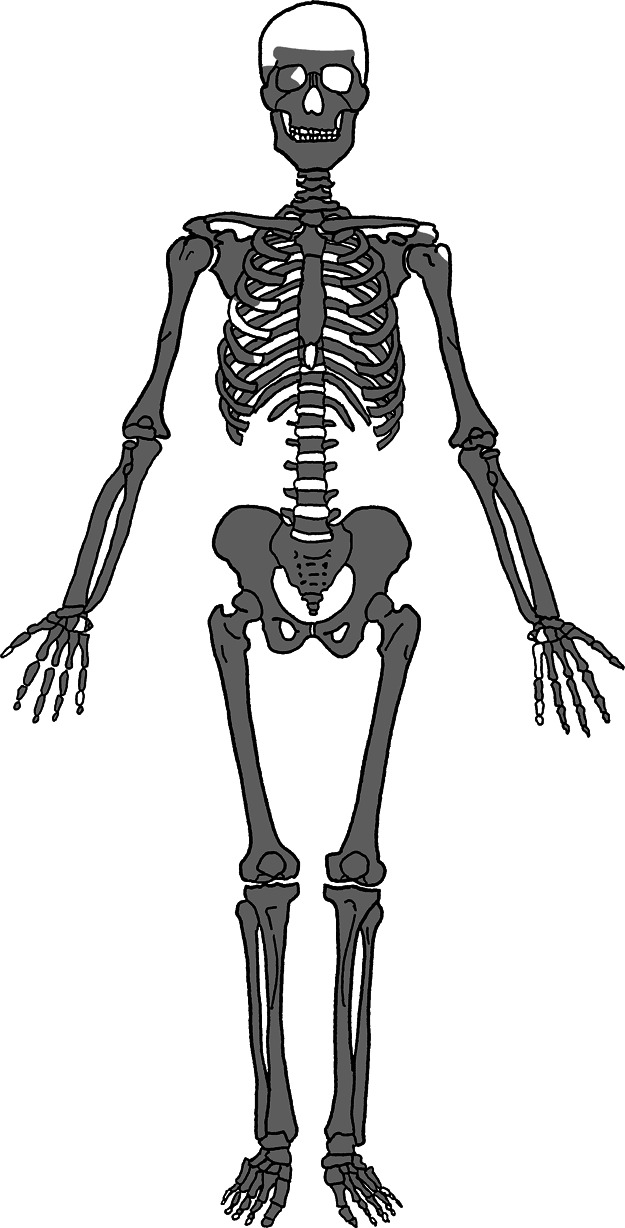
State of preservation of this case (white: unidentified or missing).

The target material of this study consisted of human remains belonging to the late Edo period excavated from the Kounji-at site, which is an Edo-period burial site in Tokyo. The tombstone indicates that this individual was a man and belonged to a high social class of *samurai* (*hatamoto*), who died at the age of 42. While the cranial vault and facial bones were damaged and some of the fifth and sixth cervical vertebrae and a few hand and foot bones were missing, the preservation of other parts was good (Figure 1). Using innominate bone morphology, we confirmed the sex (Buikstra and Ubelaker, 1994; Klales et al., 2012) and estimated age (Sakaue, 2006). The morphology of the innominate bone was most likely male in all sex estimation parts, and the estimated age was middle age (Phase IV of the Suchey–Brooks pubic symphysis scoring system). The estimated sex and age were consistent with the tombstone.

**Figure 2.**
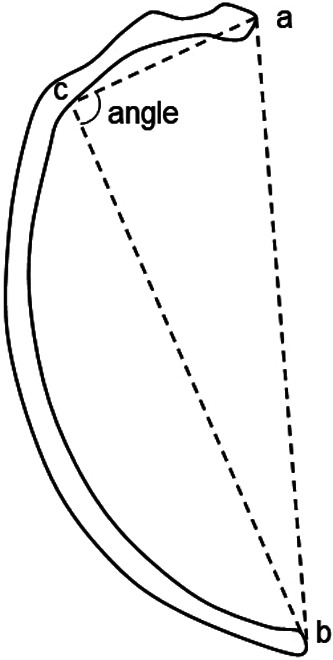
The method for measuring the angle at the costal angle: (a) the most medial protruding point of the rib head; (b) the midheight point of the visceral surface of the rib sternal end; and (c) the midheight point of the visceral surface of the costal angle.

The individual was evaluated by macroscopic observation and morphometric analysis. Measurements of the mandible, limb bones, and ribs were conducted according to Martin’s method (Baba, 1991; Martin and Saller, 1957), using a digital caliper, osteometric board, and tape measure. In addition, the straight length of the ribs (Martin No. Rib-4), and the angle at the costal angle were measured according to the following definition: (a) the most medially protruding point of the rib head; (b) the midheight point of the visceral surface of the rib sternal end; and (c) the midheight point of the visceral surface of the costal angle. After measuring the distances a–b, a–c, and b–c, the costal angle was calculated (Figure 2). The costal angle was determined at the attachment points of the deep back muscles. The second and seventh ribs, which were undamaged on both sides, were selected for the rib measurements. These measurements were compared for left–right differences and to those of ten males without lesions excavated from the same site. Additionally, the estimated stature was calculated from the maximum length of the femur using Fujii’s equation (Fujii, 1960).

After measurements and photography, the thoracic and lumbar vertebrae, sacrum, rib, and sternum were joined using an adhesive (Cemedine) and photographed from various planes. A digital camera (EOS Kiss X7, Canon) was used to take the photographs. The Cobb angle was measured from anterior-view photographs using ImageJ (NIH) image-processing software. The Cobb angle was determined by extending the inclinations of the upper and lower vertebrae in a straight line and measuring the angle at which these lines intersect; this is the same as the method used in X-ray imaging (Cobb, 1948). The kyphotic angle of the spine was measured from lateral-view photographs using the same method as that for the Cobb angle (Koelé et al., 2020). The angle of trunk rotation (ATR) was measured using a scoliometer (Ribto) (Bunnell, 1984). Clinically, this test is performed with the patient in a forward bending position. As an alternative method for measuring this individual, measurements were taken at the most prominent part of the rib cage hump.

We used computed tomography (CT, Supria, Fujifilm Corporation) to capture the structure of the spine. The scan parameters were as follows: tube voltage, 100 kVp; tube current, 175 mA; rotation time, 1.5 seconds; collimation, 0.625 mm × 64; pitch factor, 0.828. The reconstruction settings for CT were: slice thickness, 1 mm; slice interval, 0.5 mm; field of view, 300 mm; matrix size, 512 × 512; reconstruction kernel, F5. We recorded the ray summation (RaySum) image and curved planar reconstruction (CPR) images. RaySum can show an image similar to a simple X-ray image obtained, and CPR is a method for reconstructing cross-sections along tortuous surfaces and curved surfaces.

## Results

**Figure 3.**
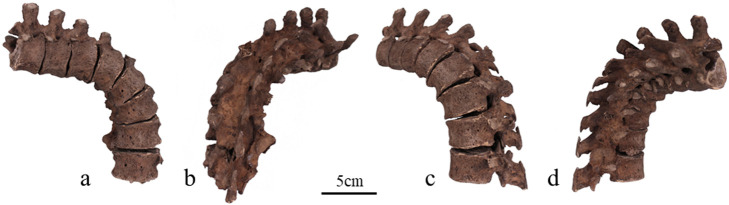
Photographs of the ankylosed thoracic vertebrae: (a) anterior view, (b) posterior view, (c) left side view, and (d) right side view.

**Figure 4.**
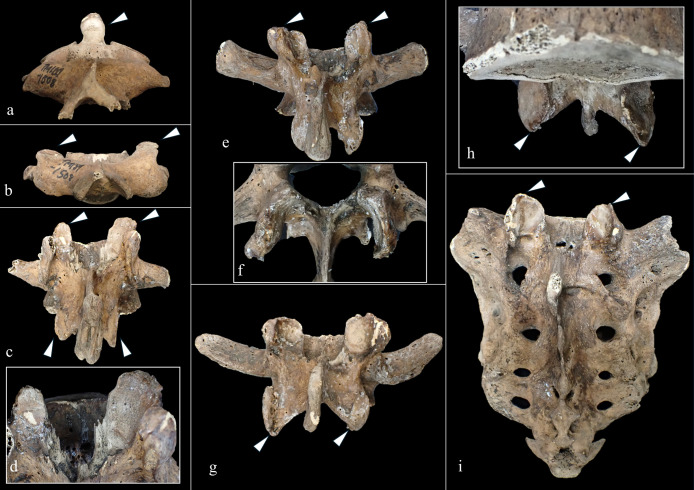
Photographs of the cervical and lumbar vertebrae: (a) axis, (b) third cervical vertebra, (c, d) first lumbar vertebra, (e, f) third lumbar vertebra, (g, h) fifth lumbar vertebra, (i) sacrum. (Arrows indicate the deformity, e.g. side difference of the superior articular process.)

**Figure 5.**
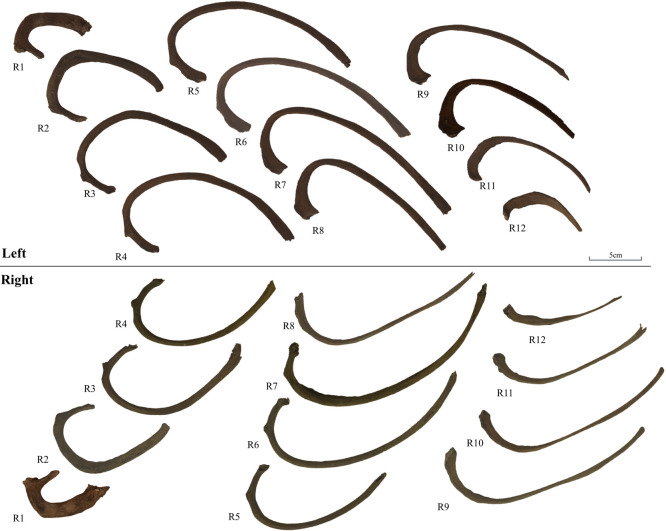
Photograph showing both sides of the rib bones.

**Figure 6.**
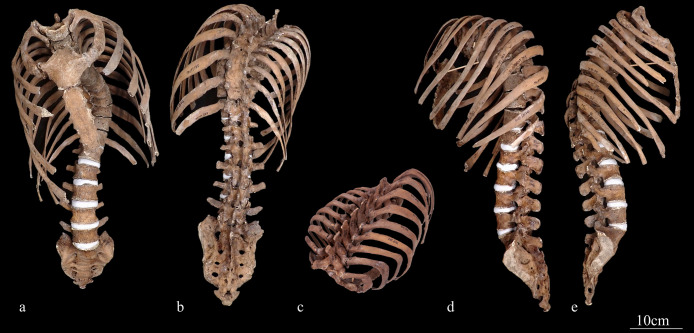
Photographs showing the joined trunk bones: (a) anterior view, (b) posterior view, (c) superior view, (d) left side view, and (e) right side view.

**Figure 7.**
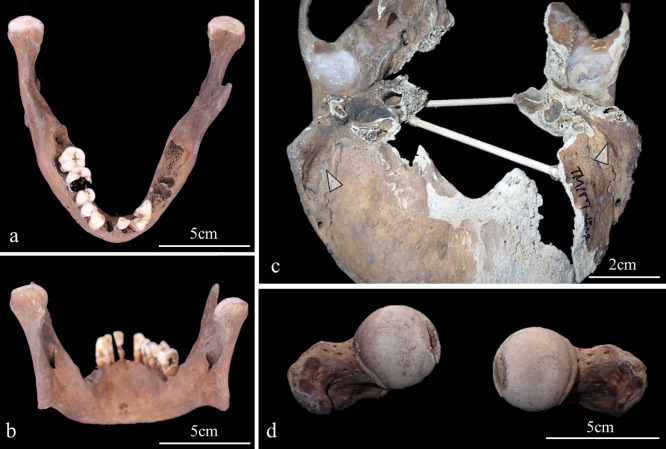
Photographs of the bone where morphological deformity was observed: (a, b) mandible, (c) inferior view of the posterior part of the cranium (arrow indicates the mastoid notch of the temporal bone), (d) difference of the femoral anteversion angle.

**Figure 8.**
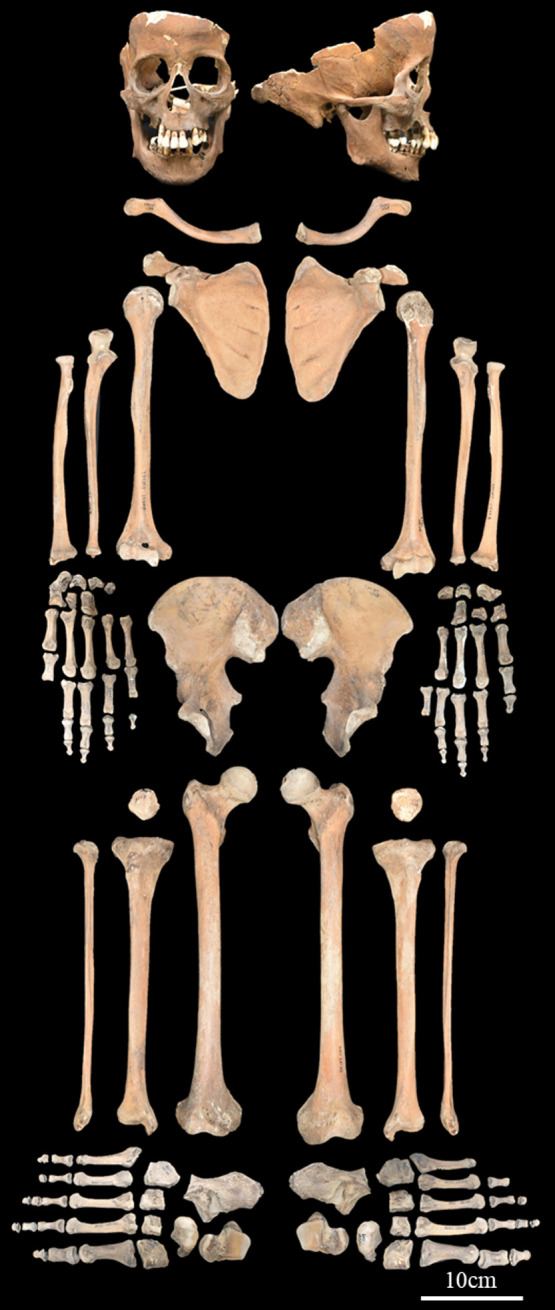
Photograph of the entire skeleton excluding trunk bones

**Figure 9.**
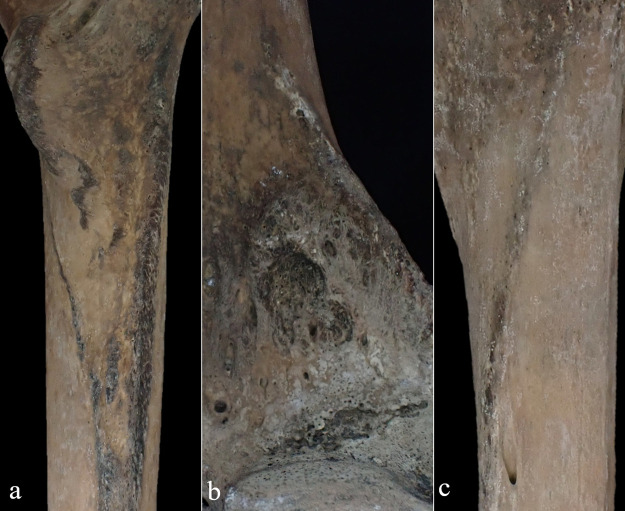
Photograph of the muscle attachment site of the lower limb (a: spiral line and gluteal tuberosity; b: origin of the gastrocnemius; c: soleal line)

**Figure 10.**
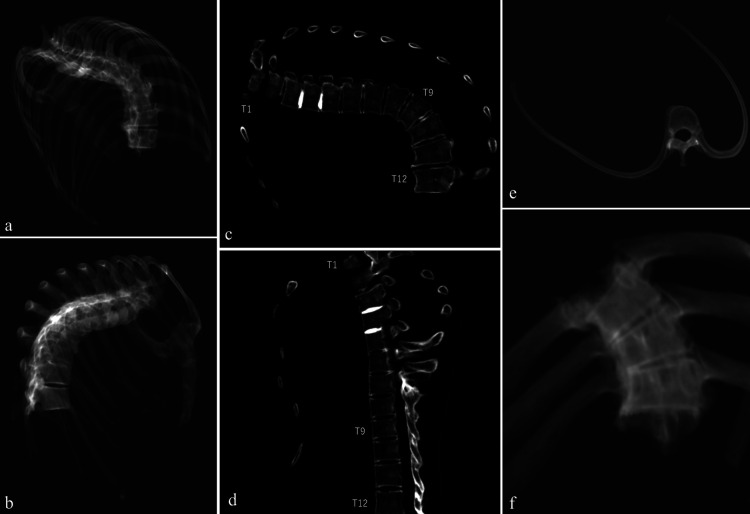
CT image of the joined thoracic bones (a, b) RaySum image of the thoracic bones (c, d) CPR image of the thoracic bones (e) upper view of the ninth thoracic vertebra and ninth rib (f) coronal plane CT scan of the eighth to tenth thoracic vertebrae.

The macroscopic observations are presented below. Figure 3 shows photographs of the individual’s ankylosed thoracic vertebrae. Ankylosis was observed in the intervertebral joints of the thoracic vertebrae from T5 to T11 on both sides, and only on the right side between T11 and T12. The ankylosed mid-to-lower thoracic vertebrae exhibited a left convex curvature with T9 as the apex vertebra, and the vertebral bodies around the apex were slightly rotated to the left, which was the convex side of the curve. Additionally, a slight decrease in the height of the vertebral bodies on the concave side compared to the convex side was noted at T8–T10. In the cervical vertebrae, the odontoid process of the axis was deviated to the left (Figure 4a), and the left superior articular process of the third cervical vertebra was displaced downward (Figure 4b). The lumbar vertebrae also exhibited deformities. The vertebral arch of the L1 and L2 was rotated to the right relative to the vertebral body, with this torsion being more pronounced in the L1 (Figure 4c). Additionally, the superior articular facet on the right side was larger than that on the left side and extended superiorly and laterally in the L1 (Figure 4d). In the L2 and L3, the superior articular facet extended to the left, and the superior articular process was inclined to the left, with this inclination being more pronounced in the L3 (Figure 4e, f). The inferior articular process of the L5 projected more inferiorly on the left than on the right (Figure 4g, h). The sacral base was inclined to the right, resulting in the superior articular process and the facet being lower on the right side than on the left side (Figure 4i). A clear asymmetry in the rib morphology was observed below the seventh rib (Figure 5). There was a noticeable asymmetry in the curvature of the mid-to-lower ribs, with the angle at the costal angle on the left and convex sides of the scoliosis being more acute than that on the right side, where the costal angle was obtuse. Additionally, the right ribs were longer than the left ribs (Figure 5). Figure 6 shows a photograph of the joined trunk bones. As shown in Figure 6c, the ribcage was elevated on the left side in the superior view of the joined trunk bones. Both mandibular rami were inclined towards the left side (Figure 7a, b). The width of the mastoid notch of the temporal bone indicated asymmetry and was wider on the right than left (Figure 7c). The right clavicle was longer than the left, and the left clavicle was more strongly curved than the right (Figure 8). The femoral anteversion angle was smaller on the left than on the right (Figure 7d). The bilateral acetabular margin exhibits marginal lipping covering more than half of the joint circumference. Figure 8 shows a photograph of the entire skeleton excluding the trunk bones, with no significant deformities other than those noted above. Muscle attachment sites such as the deltoid tuberosity of the humerus, spiral line and gluteal tuberosity of the femur, and soleal line have been developed (Figure 8, Figure 9). Figure 10 shows CT images of the thorax. The aforementioned deformities can also be observed in the CT images.

Next, the measurement results are presented. The Cobb angle was measured, with T6 as the upper-end vertebra and T11 as the lower-end vertebra, resulting in a Cobb angle of 77°. The kyphotic angle measured using the same criteria as the Cobb angle was 76°. The ATR, an indicator of the rib cage hump, was the largest at the T7 level, measuring 39°. The measurements of the mandible, limb bones, and ribs of the individuals are listed in Table 1. The straight lengths of the second rib were 73.1 mm and 74.8 mm on the right and left sides, respectively. The same measurements for the seventh rib were 191.2 mm on the right side and 161.9 mm on the left side, with the right side being 29.3 mm longer. The angle at the costal angle was 56° on the right side and 55° on the left side for the second rib, and 75° on the right side and 58.9° on the left side for the seventh rib, showing a noticeable side difference in the seventh rib. The average angle at the costal angle for Edo period male skeletons was 56.7 ± 3.7° on the right side and 55.3 ± 7.0° on the left side for the second rib, and 81.5 ± 7.4° on the right side and 83.5 ± 8.8° on the left side for the seventh rib. Regarding the measurements of the limb bones, the maximum length of the clavicle was 138 mm on the right side and 118 mm on the left side, with the right side being 20 mm longer (Figure 3e, f). The angle of torsion of the femur (Martin No. Femur-28) which relates to the femoral anteversion angle was smaller on the right than the left (right, 6°; left, 19°). No significant asymmetry was observed in the other limbs. The length–thickness index, which is the ratio of the diaphyseal circumference and bone length, of the humerus and femur was 18.3 on the right side, 17.9 on the left side, and 18.4 on the right side, 17.7 on the left side, respectively, which were close to the lower limit of the distribution for male skeletons from the same site (right humerus, 19.8 ± 1.3; right femur, 20.8 ± 1.2), indicating a slender circumference relative to the maximum length of both upper and lower limbs. The midshaft indexes of the femur (right, 102.3; left, 105) and tibia (right, 73.8; left, 72.5) were not different from the average of the same site male (right femur, 106.9 ± 7.4; right tibia, 74.1 ± 5.4). The estimated stature of this individual, calculated from the maximum length of the femur using Fujii’s equation, was 150.3 cm.

## Discussion

In modern clinical practice, a Cobb angle of 10° or more is considered scoliosis, and an angle of 45° or more is considered severe scoliosis, which warrants surgical intervention (Cheng et al., 2015; Weinstein et al., 2008). The Cobb angle of the individual in this study was 77°, which exceeded the threshold for severe scoliosis. Additionally, the ATR, an indicator of the rib cage hump, was 39° on the left side, significantly exceeding the clinical suspicion threshold of 5° for scoliosis (Bunnell, 1984). In terms of thoracic deformities due to scoliosis, it has been shown that when the convex side deforms dorsolaterally, the ribs in the concave side deform medially. Furthermore, scoliosis results in an asymmetry in rib length, with ribs in the concave side being longer than ribs in the convex side. The rib deformities in this individual were consistent with those reported to be associated with scoliosis (Schlager et al., 2022; Zhu et al., 2011). Moreover, the normal range for thoracic kyphosis is 20°–40° (Roaf, 1960), with 50° or more being considered hyperkyphosis (Koelé et al., 2020). In this case, the kyphotic angle of the thoracic spine was 76°, which significantly exceeded the threshold. Scoliosis and kyphosis of the thoracic spine are structural deformities that are accompanied by bony ankylosis. Moreover, the deformity of the first and second lumbar vertebrae is suggested to be related to the major thoracic curve. Additionally, in the cervical vertebrae, there was a leftward deviation of the odontoid process of the axis, and a downward deviation of the left superior articular facet of the third cervical vertebra. In the fifth lumbar vertebra and sacrum, there was a side difference in the articular process and an inclination of the sacral base. Thus, the individual’s spine exhibited a triple curve with the major structural curve in the midthoracic to upper lumbar spine and a compensatory minor curve in the cervical and lower lumbar spine. According to the clinical classification criteria by Lenke et al. (2002), the curve type is classified as type 1 because of the main thoracic curve. The lumbar modifier can be determined as type B. This is because the vertical line from the sacrum did not pass through the spinous processes of the apex of the lumbar curve. Additionally, even considering the misalignment during joining, it is unlikely that the vertical line from the sacrum would pass through the medial side of the apex of the lumbar curve. The sagittal thoracic modifier was positive when the kyphosis angle exceeded 40°, classifying it as 1B+. In conclusion, this individual had severe structural kyphoscoliosis.

Diseases manifesting with structural scoliosis, such as this individual, can be categorized as idiopathic scoliosis, congenital scoliosis, and neuromuscular scoliosis caused by conditions such as cerebral palsy, muscular dystrophy, syringomyelia, and mesenchymal tissue disorders (e.g. Marfan syndrome). Representative diseases presenting with kyphosis include Scheuermann’s disease, whereas neurofibromatosis can present with kyphoscoliosis. Appleby et al. (2014) recommended that in the differential diagnosis of scoliosis in archaeological skeletal remains, specific diseases should first be ruled out, and if none of these diseases match, idiopathic scoliosis should be considered. This is also the same in clinical practice (Weinstein et al., 2008). In this study, we used a differential diagnostic method to estimate the disease in this case.

A comparative examination of the characteristics of diseases other than idiopathic scoliosis is presented below. Congenital scoliosis is caused by congenital abnormalities in the spine. This condition results in segmentation abnormalities and malformed vertebrae such as hemivertebrae, butterfly vertebrae, and wedge vertebrae, none of which were observed in this case, ruling out congenital scoliosis (Mackel et al., 2018). Marfan syndrome, in addition to scoliosis, is characterized by tall stature, long limbs, and arachnodactyly (Dean, 2007). The estimated stature of this individual, calculated from the maximum length of the femur, is 150.7 cm, which is less than other males from the same site (155.0 ± 5.4 cm). Additionally, there were no abnormalities in the proportions of the limbs which did not align with the characteristics of Marfan syndrome. Neuromuscular scoliosis arises from conditions, such as cerebral palsy, Duchenne muscular dystrophy, and syringomyelia. Cerebral palsy affects patients in various ways, with 70–80% exhibiting spastic paralysis and 10–20% presenting with athetosis or dyskinesia. It has been reported that patients with spastic paralysis frequently develop scoliosis (Gulati and Sondhi, 2018; Saito et al., 1998). A previous study had documented archaeological cases of severe scoliosis diagnosed with cerebral palsy with right-sided hemiplegia (Tesorieri, 2016). In this case, no atrophy of unilateral limbs, as indicated in the previous research, has been observed. Clinical studies have shown that the severity of scoliosis in individuals with cerebral palsy is associated with severe motor function impairments, such as gait disturbances. It has also been reported that scoliosis rarely becomes severe in cases of mild motor function impairments (Persson-Bunke et al., 2012). In our case, the muscle attachment sites of the upper and lower limbs were well developed without significant asymmetry, suggesting that there were no severe motor impairments such as notable gait disturbances. Therefore, there are no strong supporting factors to confirm that this case was cerebral palsy. Patients with Duchenne muscular dystrophy typically die in their late teens to early thirties (Posky, 2007), which does not match the age at death of this individual (42 years). Neurofibromatosis type 1 frequently presents with spinal deformities such as kyphoscoliosis (Lewis, 2019). Spinal deformities are classified into dystrophic and non-dystrophic types. In dystrophic type, erosive lesions such as scalloping of the vertebral bodies, thinning of the transverse processes, and penciling of the ribs occur (Winter et al., 1979). Erosion or infiltration of bone by localized neurofibromas has been suggested as a factor in the development of spinal deformities (Funasaki et al., 1994). In this case, no deformities characteristic of the dystrophic type of spinal deformity were observed, thus not supporting the diagnosis of dystrophic type scoliosis of the neurofibromatosis. Non-dystrophic scoliosis presents with deformities similar to idiopathic scoliosis, making differentiation from idiopathic scoliosis difficult. Scheuermann disease typically presents as kyphosis. The diagnostic criteria for this disease include anterior wedging of three or more consecutive vertebral bodies; however, no anterior wedging of the vertebral bodies was observed in this case, ruling this out. Syringomyelia presents with spinal deformities similar to those in idiopathic scoliosis. The left curved scoliosis observed in this individual is atypical for idiopathic scoliosis and is more common in syringomyelia (Qiu et al., 2008; Wu et al., 2010). Therefore, in modern clinical practice, when such an atypical spinal curvature is observed, magnetic resonance imaging (MRI) is recommended to differentiate between conditions such as syringomyelia or idiopathic scoliosis (Wu et al., 2010). However, clinically differentiating syringomyelia from idiopathic scoliosis requires MRI of the spinal cord; in this case, it was impossible to differentiate between syringomyelia and idiopathic scoliosis based solely on bone morphology. Moreover, none of the previously mentioned diseases are typically characterized by bony ankylosis. The ankylosis observed in the mid to lower thoracic vertebrae in this case is presumed to be associated with immobility.

Examination of several differential diagnoses for spinal deformity in this case revealed that the patient likely had either idiopathic scoliosis or syringomyelia. In addition to visible changes, the symptoms associated with severe scoliosis include lower back pain, back pain, and respiratory symptoms. When the thoracic Cobb angle is greater than 50°, a decrease in lung capacity due to reduced thoracic volume can be observed (Weinstein et al., 2008). Given the severity of kyphoscoliosis in this case, the patient may have experienced respiratory dysfunction. However, well-developed muscle attachment sites throughout the body suggested that the individual was not bedridden or extremely immobile. As indicated in several paleopathological texts, reports of scoliosis in skeletal remains from archaeological sites are rare, and this case is likely the first detailed report of the archaeological case excavated from the Japanese Archipelago. Despite the atypical left convex curvature, the individual presented typical bone morphological abnormalities associated with scoliosis, making this a significant example for the study of scoliosis in skeletal remains.

## Figures and Tables

**Table 1.** Measurements (mm, degrees) of mandible, rib, and limb bones

Martin’s No.	Variable	Right	Left
Mandible			
66	Bigonial breadth	93	
69	Symphyseal height	35	
70	Ramus height	64	66
71	Ramus breadth	36	34
Clavicle			
1	Maximum length	138	118
Humerus			
1	Maximum length	273	280
5	Maximum midshaft diameter	19	19
6	Minimum midshaft diameter	14	13
7	Minimum circumference	50	50
Radius			
1	Maximum length	234	233
4a	Transverse midshaft diameter	11	10
5a	Sagittal midshaft diameter	11	9
5(5)	Midshaft circumference	34	33
Ulna			
1	Maximum length	207	207
3	Least circumference	34	33
11	Transverse shaft diameter	11	10
12	Sagittal shaft diameter	11	9
Rib			
4	Straight length of limb–2nd rib	73	75
	Angle at the costal angle–2nd rib	56	55
4	Straight length of limb–7th rib	191	162
	Angle at the costal angle–7th rib	75	59
Femur			
1	Maximum length	386	390
6	Sagittal midshaft diameter	22	21
7	Transverse midshaft diameter	22	22
8	Midshaft circumference	71	69
Tibia			
1a	Maximum length	313	317
8	Anteroposterior midshaft diameter	24	23
9	Mediolateral midshaft diameter	18	17
10	Midshaft circumference	67	63
Fibula			
1	Maximum length	307	311
4a	Minimum circumference	34	34
